# Supplementary material for: Rational Engineering of (S)-Norcoclaurine Synthase for Efficient Benzylisoquinoline Alkaloids Biosynthesis
Source: Molecules. 2023 May 23;28(11):4265. doi: 10.3390/molecules28114265 (PMC10254637; doi:10.3390/molecules28114265)
Supplement: Supplementary file 1 [file molecules-28-04265-s001.zip › molecules-2388217-supplementary.pdf]

Rational Engineering of (*S*)-Norcoclaurine Synthase for Efficient Benzylisoquinoline Alkaloids Biosynthesis

João P. M. Sousa, Nuno C. S. A. Oliveira, Pedro A. Fernandes*

LAQV/REQUIMTE, Departamento de Química e Bioquímica, Faculdade de Ciências Universidade do Porto, Rua do Campo Alegre, s/n, 4169-007 Porto, Portugal

E-mail: pafernan@fc.up.pt

SUPPORTING INFORMATION

STRUCTURE VALIDATION

The Ramachandran analysis of the built model shows that 97 % of the residues are in the favored regions (light blue regions, see Figure S1), and there is only one residue slightly away from the allowed regions (dark blue regions, see Figure S1-C). The residue in question (Thr148) is a prePro residue and is part of a small *β*-turn far away from the active site. Its position in the Ramachandran plot was not seen as problematic, and it is not expected to disturb the system during further simulations.

The tertiary structure evaluations also show results strongly in favor of our model. The scores from QMEAN[1] (score: -0.51, see Figure S2-A) and ProSaII[2] (Z-score: -5.88, see Figure S2-B) show that the protein’s folding is perfectly comparable to the ones observed for native structures of identical size, highlighting a successful modeling task

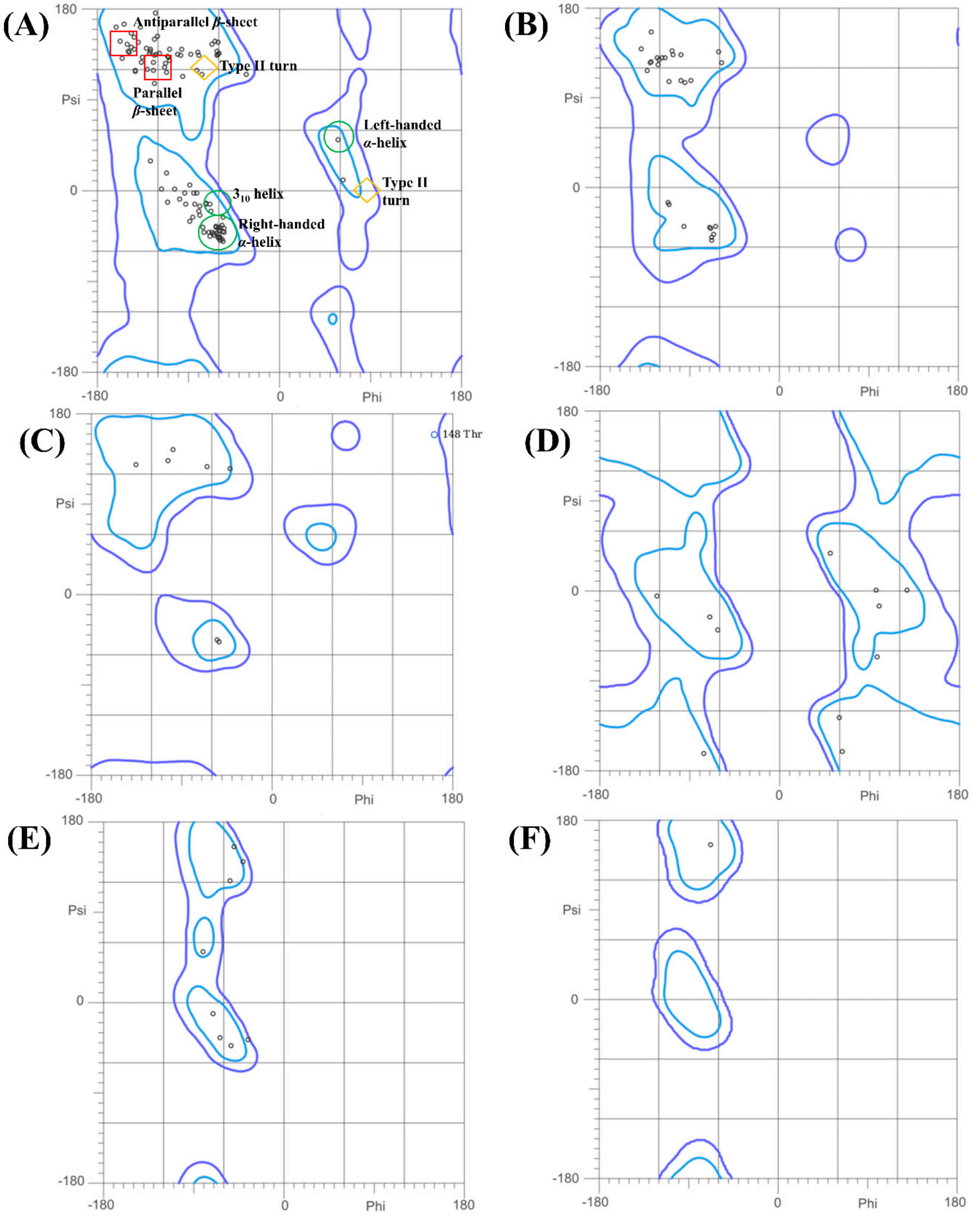

Figure S1: The Ramachandran plot for the constructed *Ps*NCS structure: (A) general graphic; (B) graphic for the Ile and Val residues; (C) graphic for the prePro residues; (D) graphic for the Gly residues; (E) graphic for the transPro residues; (F) graphic for the cisPro residues. In graphic (A), α-helix regions are identified with green circles, β-sheet regions with red squares, and type II turn regions with yellow diamonds.

.

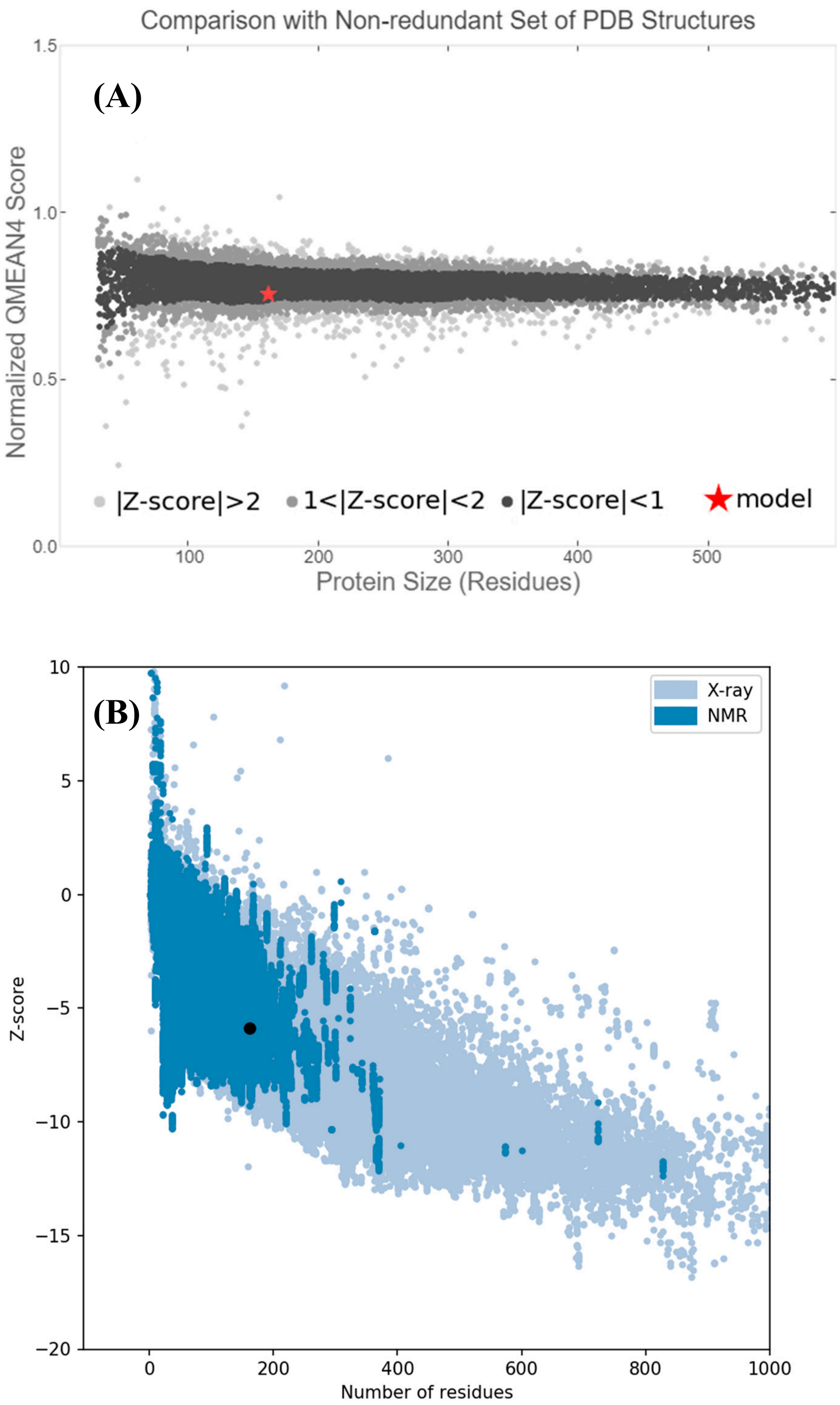

Figure S2: The tertiary structure comparison plots, using: (A) QMEAN[26,33]; (B) ProSaII[34]. In graphic (A), the QMEAN4 score used for evaluation results from the linear combination of four statistical potential terms. It is transformed into a Z-score to relate it with what is expected from high-resolution X-ray structures.[33] In graphic (B), the black dot represents the model's location in the plot.

MOLECULAR DOCKING OUTPUT

The docking procedure yielded four poses (A to D, see Figure S3) which the software scored as follows: A > B > C > D. Taking the reaction's mechanistic data into account, we discarded the poses C and D because they had the aldehyde moiety interacting with Lys121 instead of the dopamine moiety. The reaction is highly unlikely to proceed from this orientation, as shown by earlier quantum chemical calculations. Poses A and B displayed a favorable orientation. To choose one of them, we inspected the most relevant interactions for the reaction: between the intermediate's carbonyl oxygen and the amino group side chain of Lys121 (Lys121-NH_3^+···O=C-QIN), and between the carboxylate side chain of Glu109 and the intermediate's carbon atom whose proton is to be abstracted (Glu109-COO^-···HC-QIN). We saw that the interaction with Lys121 was comparable for both poses (2.32 Å for A / 2.47 Å for B), but B showed a favored interaction with Glu109, compared with A (5.03 and 5.38 Å, respectively). We noted that B was consistent with a productive conformation for dopamine upon binding, whereas A was related to a less productive one. This slight difference is significant because it changes the position of the aldehyde's R-group in the active site. The dopamine conformation of B maintains the aldehyde closer to the active site's entrance, while in A, it is forced towards the active site's center. The consequence is the concealment of the carbon atom that should be close to Glu109. Considering this, we decided that B should be the more suitable complex to progress for the following stages, even though both binding modes are predicted to favor the course of the reaction.

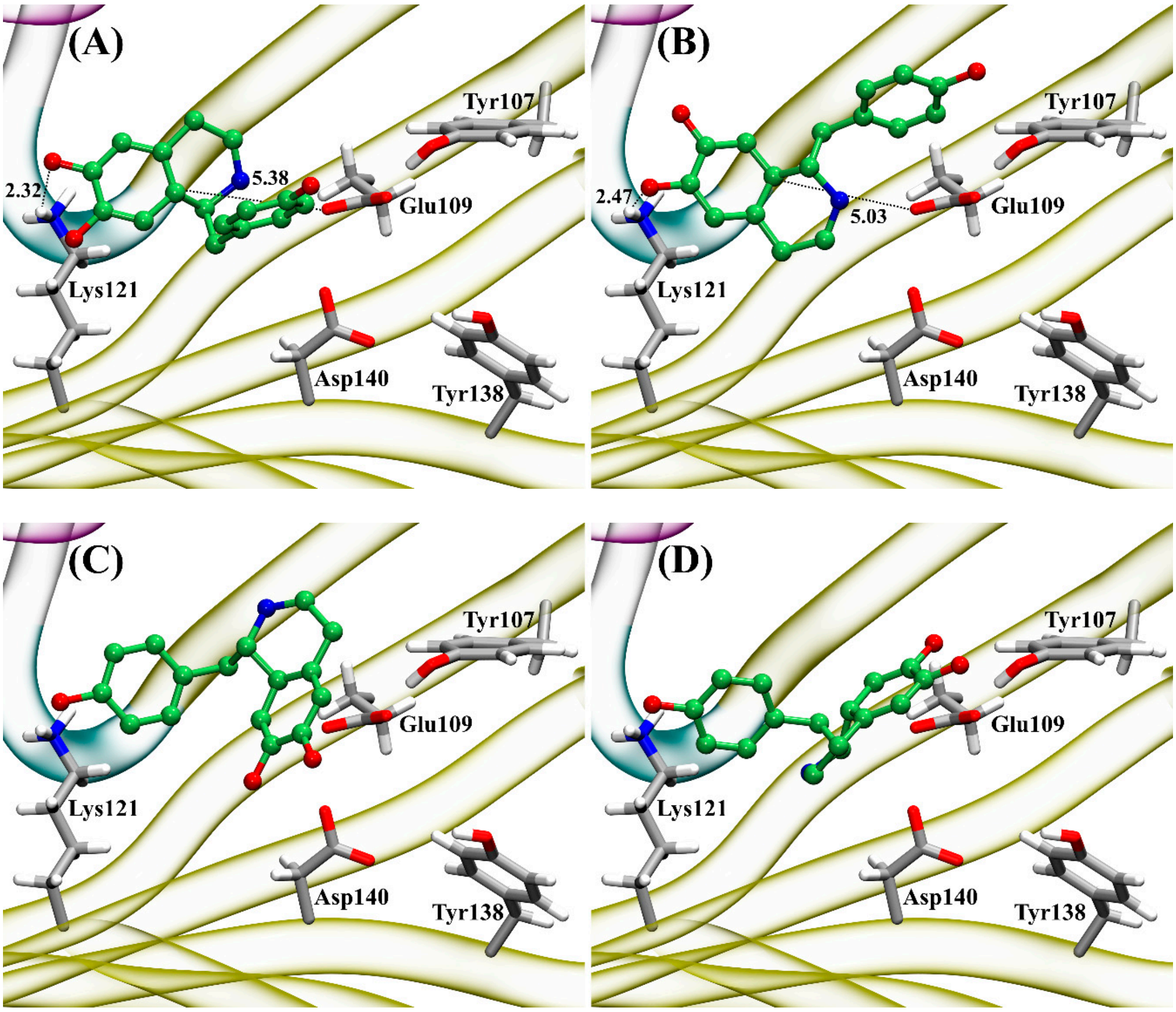

Figure S3: The active site of NCS in complex with the four poses of QIN, resulting from molecular docking. The inspected interactions for A and B are highlighted with dotted lines, and the numbers denote the distance in Å.

MOLECULAR DYNAMICS SIMULATIONS

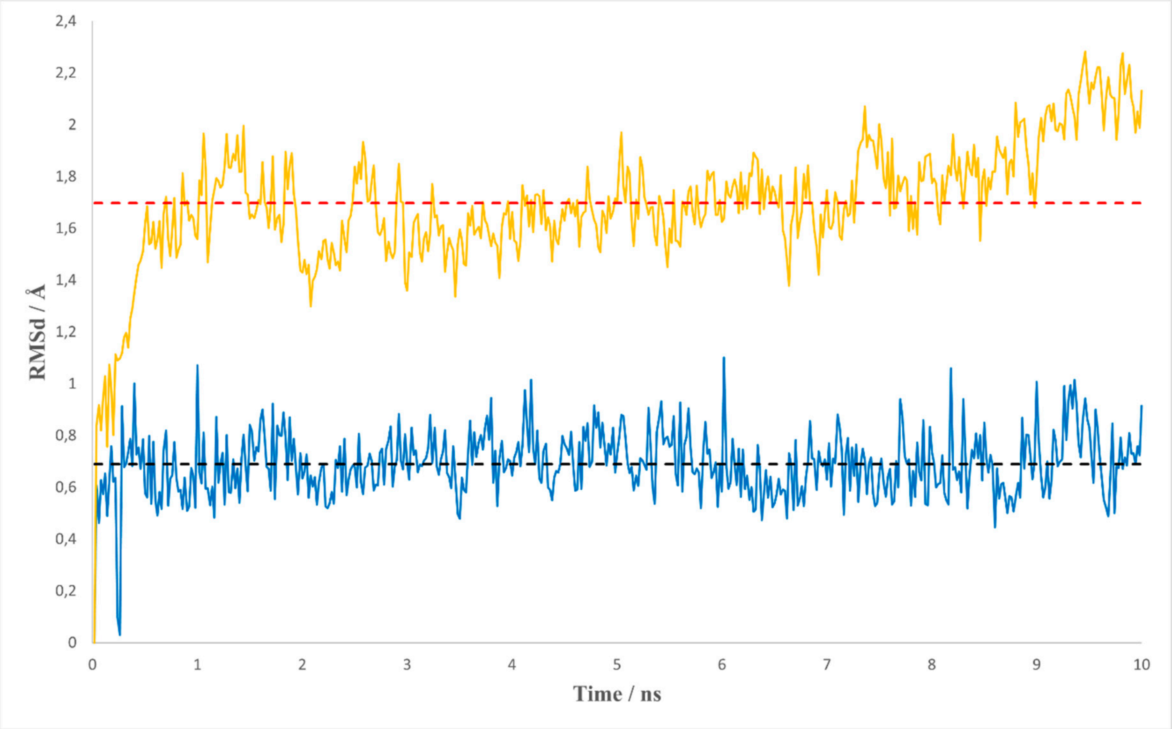

Figure S4: The RMSd values calculated for the protein backbone (yellow line) and the active site (blue line) of the system throughout the production stage of the MD simulation. For the active site RMSd calculation, we considered the catalytic water, the QIN intermediate, and the residues Tyr107, Glu109, Lys121, and Asp140. The black dashed line indicates the average deviation calculated for the active site (0.69 Å), and the red one refers to that value for the protein backbone (1.70 Å).

REACTION STATIONARY POINTS

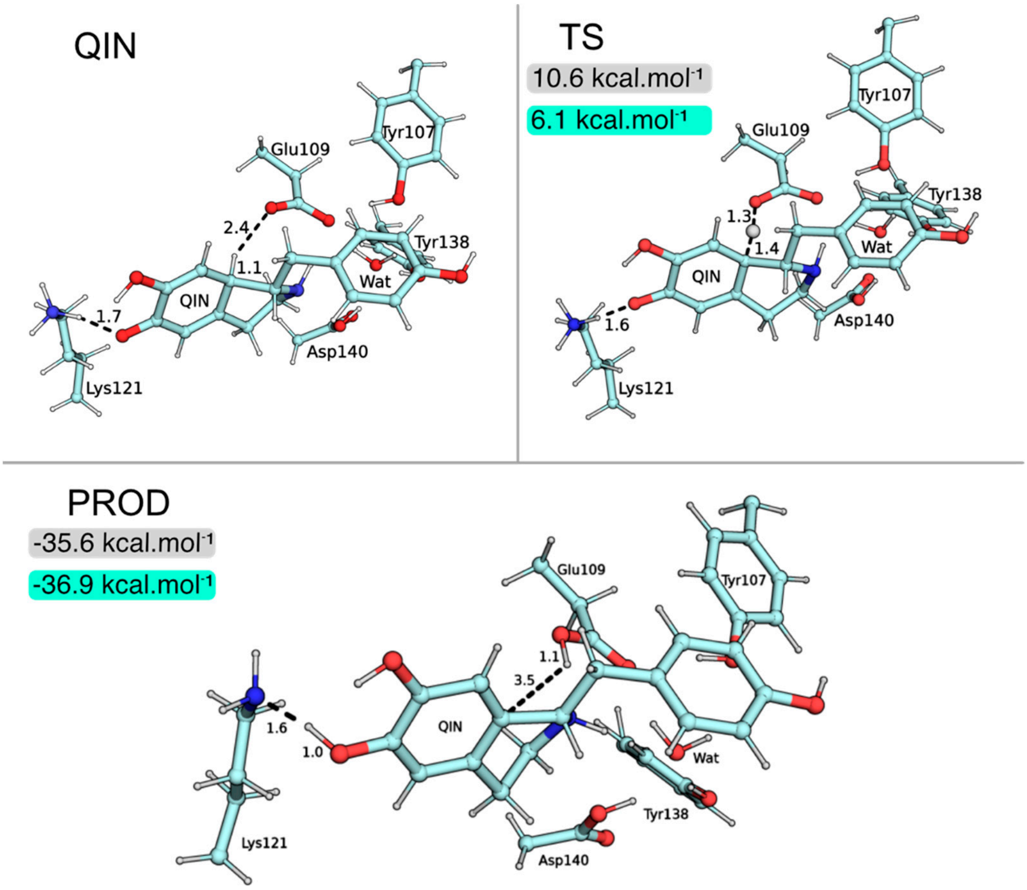

Figure S5: Stationary points geometries of the rearomatization step respective $\Delta E^{\ddagger}$ (grey, obtained from single-point energy calculations) and $\Delta G^{\ddagger}$ (green, with added Zero-point, thermal, and entropic corrections).

[1] Benkert, P.; Tosatto, S. C.; Schomburg, D., Qmean: A Comprehensive Scoring Function for Model Quality Assessment. *Proteins: Structure, Function, and Bioinformatics* **2008**, *71*, 261-277.

[2] Wiederstein, M.; Sippl, M. J., Prosa-Web: Interactive Web Service for the Recognition of Errors in Three-Dimensional Structures of Proteins. *Nucleic acids research* **2007**, *35*, W407-W410.
